# Circumventing huge volume strain in alloy anodes of lithium batteries

**DOI:** 10.1038/s41467-020-15452-0

**Published:** 2020-04-13

**Authors:** Hongyi Li, Takitaro Yamaguchi, Shingo Matsumoto, Hiroaki Hoshikawa, Toshiaki Kumagai, Norihiko L. Okamoto, Tetsu Ichitsubo

**Affiliations:** 1 Institute for Materials Research, Tohoku University, Sendai, 980-8577 Japan; 2 Advanced Materials Development Laboratory, Sumitomo Chemical Co., Ltd., Tsukuba, 300-3294 Japan; 3 Energy and Functional Materials Research Laboratory, Sumitomo Chemical Co., Ltd., Ehime, 792-8521 Japan

**Keywords:** Batteries, Metals and alloys

## Abstract

Since the launch of lithium-ion batteries, elements (such as silicon, tin, or aluminum) that can be alloyed with lithium have been expected as anode materials, owing to larger capacity. However, their successful application has not been accomplished because of drastic structural degradation caused by cyclic large volume change during battery reactions. To prolong lifetime of alloy anodes, we must circumvent the huge volume strain accompanied by insertion/extraction of lithium. Here we report that by using aluminum-foil anodes, the volume expansion during lithiation can be confined to the normal direction to the foil and, consequently, the electrode cyclability can be markedly enhanced. Such a unidirectional volume-strain circumvention requires an appropriate hardness of the matrix and a certain tolerance to off-stoichiometry of the resulting intermetallic compound, which drive interdiffusion of matrix component and lithium along the normal-plane direction. This metallurgical concept would invoke a paradigm shift to future alloy-anode battery technologies.

## Abstract (web summary)

Alloy anode materials in lithium batteries usually suffer from fatal structural degradation due to the large volume change during cycling. Here the authors report a design in which Al foil serves as both anode and current collector to circumvent the strain.

## Introduction

Rechargeable batteries are indispensable devices in modern society and they are continuously improved toward higher energy density and longer lifetime^1,2^. In lithium-ion batteries (LIBs) as a representative rechargeable battery, the combination of intercalation-type transition-metal-oxide cathode and carbonaceous anode materials have achieved a great success and win the current energy-storage device market^3–5^. However, as the energy density of conventional LIBs have gradually reached the theoretical limit, further approaches are necessary for developing superior batteries^6,7^. As a promising approach, high-capacity alloy-anode materials, such as Si, Sn, or Al, had been expected for the practical applications in advanced lithium batteries because of their much (three to ten times) higher capacities than a conventional graphite anode^6,8–11^. Compared with the intercalation-type graphite anode^12,13^, the alloy anodes must suffer large volume change during battery reactions^11,14,15^, e.g., +300% volume expansion (increase) from Si to Li_21_Si_5_ (~4000 ${\mathrm{mAh}}\;{\mathrm{g}}_{{\mathrm{Si}}}^{ - 1}$), +250% from Sn to Li_22_Sn_5_ (~ 990 ${\mathrm{mAh}}\;{\mathrm{g}}_{{\mathrm{Sn}}}^{ - 1}$), and +100% from Al to AlLi (~ 990 ${\mathrm{mAh}}\;{\mathrm{g}}_{{\mathrm{Al}}}^{ - 1}$), compared with +10% volume expansion from C_6_ to LiC_6_ (372 ${\mathrm{mAh}}\;{\mathrm{g}}_{\mathrm{C}}^{ - 1}$). Such a huge volume change accompanied by lithiation or delithiation is substantially isotropic and causes critical irreversible damage to the matrix, which would consequently be fragmented into inactive fine powder drifting in the electrolyte^16–18^. Therefore, if a metallic foil (especially, Sn or Al) could maintain a stable electrode structure during battery reactions, a heavy Cu current collector currently used would have been unnecessary, which would have benefited a remarkable increase of practical anode capacity. However, the application of alloy anodes has never been succeeded yet. One of effective approaches to avoid this pulverization problem is to downsize particles of active material and/or to fabricate porous electrode morphology, by which the volume change can be tolerated owing to the voids between the particles^19–26^. However, the active materials must be prepared with supporting substances, such as in the form of composite electrode^27–29^ or attaching on nanostructures^30–35^. Preparation of such supporting materials usually makes the manufacturing process complicated and decreases the practical anode capacity, which has impeded the development of alloy anodes.

Nevertheless, application of alloy anodes is still strongly demanded for easy-to-manufacture strategy of large-scale LIBs. In this work, to establish a concept of “one-material electrode,” which combines active material and collector, we show an innovative way of how to circumvent such a huge volume strain by utilizing metallurgical viewpoints such as thermodynamics, elastic strain, and diffusion. By examining structural stabilities of various metallic-foil electrodes during lithiation, we demonstrate that Al foil with appropriate hardness can be used as a self-standing anode for lithium batteries. An appropriate hardness of the matrix and a certain tolerance to off-stoichiometry of the resulting intermetallic compound can drive a two-dimensionally (in-plane) homogeneous lithiation reaction and one-dimensional (out-of-plane) interdiffusion to hold the Gibbs–Duhem relation, which effectively circumvent the volume-strain influences during battery reaction and the fatal pulverization problem of alloy anodes.

## Results

### Morphology after initial lithiation into various metals

**Fig. 1:**
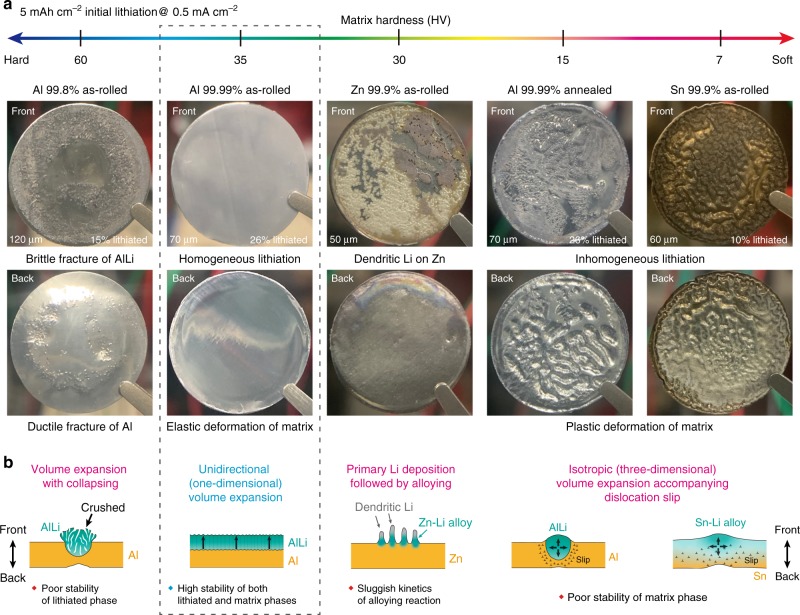
Structural stability of various metallic foils after initial lithiation. **a** Photographs of various metallic foils after lithiation of 5 mAh cm^−2^. Average Vickers hardness (HV) of each foil is given in the upper graph. The lithiation was conducted at a current density of 0.5 mA cm^−2^ with a Li metal counter-electrode. The original thickness and lithiation percentage of each foil are also denoted in the front-side photo. The percentage figure of lithiation in each sample is displayed based on the total capacity of each anode depending on the foil thickness. As to Zn, the percentage is not presented, because Li metal is mainly deposited on the surface instead of the formation of Li-Zn alloy; see also Supplementary Fig. 1c. The as-rolled Al (99.99%) foil shows a homogeneous lithiation on the front side and a sufficiently retained stable Al matrix on the back side. **b** Schematic illustrations of lithiation process reasonably inferred from SEM pictures obtained for respective foils; see Supplementary Fig. 1.

To examine the structural stability of metal foil anodes, Al, Zn, and Sn foils were lithiated in coin-type cells with a Li counter-electrode, in which the electrolyte consisting of 1 M LiPF_6_ and Ethylene Carbonate (EC) : Dimethyl Carbonate (DMC) in an equivolume ratio (50:50) was used for all the electrochemical tests. In general, the hardness of materials should largely affect the structural stability after lithiation. Thus, as to Al foils, we prepared three types of samples with different hardness, by modifying purity and controlling conditions of rolling and heat treatment. Vickers hardness (HV) of each foil is shown in Fig. 1a. The foils were lithiated to a cathodic charge of 5 mAh cm^−2^ at the current density of 0.5 mA cm^−2^. As shown in Fig. 1a, the lithiated foils have distinctively different appearances.

**Fig. 2:**
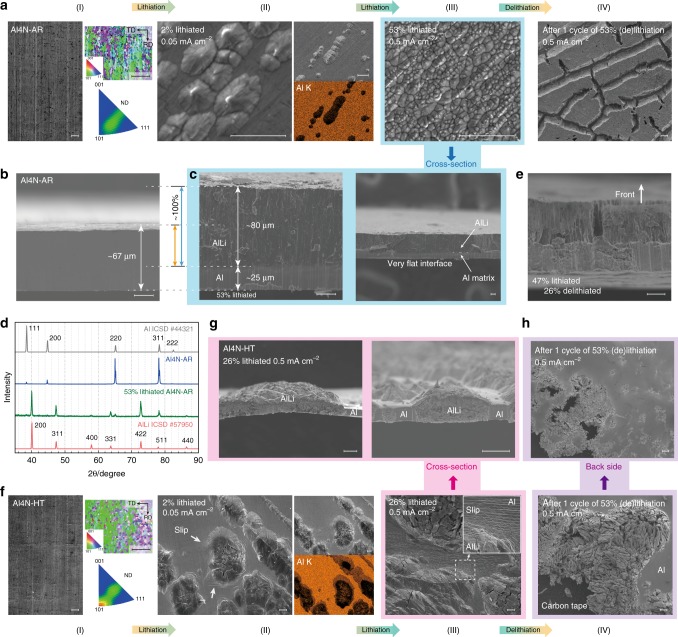
Lithiation–delithiation processes of Al4N-AR (purity 99.99%, as-rolled Al) foil and Al4N-HT (purity 99.99%, well-annealed Al) foil. **a** Morphology changes (I, II, II, and IV) on the front side (facing the Li electrode) of Al4N-AR during lithiation and delithiation, and EBSD pattern of the initial state. Al4N-AR shows a homogenous lithiation and maintains a stubborn Al-matrix layer with a good structure stability. **b** Cross-section of a bare Al4N-AR foil. **c** Cross-section of a 53%-lithiated Al4N-AR foil. A very flat interface is observed between lithiated phase and Al matrix. The lithiated layer becomes twice thick, suggesting that the volume expansion occurs along the direction normal to the Al foil (i.e., one-dimensionally). **d** XRD profiles obtained in the reflection geometry for the Al4N-AR foil before and after lithiation. **e** Cross-section of an Al4N-AR foil that has undergone 47% lithiation and subsequent 26% delithiation. Al matrix is self-organized to form a columnar structure during delithiation. **f** Morphology changes (I, II, III, and IV) on the front side of Al4N-HT during lithiation and delithiation, and EBSD pattern of the initial state. The AlLi grains are inhomogeneously formed, resulting in the pulverization during delithiation. **g** Cross-section of a 26% lithiated Al4N-HT foil, where the AlLi phase penetrates the foil accompanied with plastic deformation. **h** Backside view of an Al4N-HT foil that has undergone a 53% lithiation and delithiation cycle. The foil is pulverized during delithiation. Scale bars in SEM images are 20 μm for **a**–**f** and 100 μm for **g**, **h**. EBSD patterns of Al matrices inset in **a** and **f** indicate a marked grain growth in the recrystallization of Al4N-HT. Scale bars in EBSD patterns are 30 μm and 1 mm for Al4N-AR and Al4N-HT, respectively. The percentage figures of lithiation are displayed based on the total capacity of the Al foil.

First, it is interesting to note that soft metals less than HV 30, Sn (purity 99.9%) foil, and annealed 4N-Al foil (purity 99.99%, hereafter termed as “Al4N-HT”), are locally deformed plastically after lithiation. Undulation of the matrix, which is formed by alloying with Li on the front side (facing a Li-metal counter-electrode), is spread out the whole matrix. Besides, the lithiation occurs inhomogeneously, which indicates that the mechanical (elastic/plastic) deformation of matrix strongly affects the lithiation process. In contrast to the soft foils, hard as-rolled 2N-Al foil (purity 99.8%) shows very different lithiation behavior. On the front side, protruding AlLi grains are cracked (the phase identification is later shown in Fig. 2c), which suggests that the growing AlLi alloy could not deform the Al matrix but was contrary crushed by the hard Al matrix. Whereas dimple patterns are observed on the back side, suggesting that ductile fracture occurs due to the large tensile stress caused by the protruding AlLi grains on the front side. The morphology details are shown in Supplementary Fig. 1a.

Most importantly and surprisingly, the as-rolled 4N-Al foil (purity 99.99%, referred to as “Al4N-AR”) achieves homogeneous lithiation on the front side and no-damaged Al matrix remains on the back side. Thus, it seems that an appropriate hardness of matrix would prevent the deformation of anode matrix and the fracture of alloy phase; in the case of Al/AlLi, the most suitable hardness is eventually found to be ca. HV 35. The fact that there is no apparent plastic deformation in the matrix means that the volume expansion during lithiation occurs in the normal (i.e., out-of-plane) direction, i.e., unidirectional growth of the lithiated phase is driven. In contrast, although as-rolled Zn (purity 99.9%) has a similar matrix hardness to Al4N-AR, Li is almost deposited on the surface of Zn foil rather than forming a Zn-Li alloy. This difference would be attributed to the diffusivity of Li in Zn and the activation energy of lithiation, etc.^36,37^. With a detailed explanation in Supplementary Note 1, scanning electron microscopy (SEM) images corresponding to Fig. 1 are given in Supplementary Fig. 1 and the potential profiles are shown in Supplementary Fig. 2 for comparison.

### Attainment of homogeneous and unidirectional lithiation

To understand the differences in the degree of homogeneity on lithiation reaction and in characteristic feature of volume expansion, we observed the morphology changes accompanied by Li insertion and extraction, the results of which are summarized in Fig. 2a–h. Figure 2a shows field-emission SEM (FE-SEM) images of Al4N-AR in the lithiation process (I, II, and III) and delithiation after lithiation (IV), and Fig. 2f shows those of Al4N-HT as well. In the early stage (I–II) of lithiation, AlLi phase forms on the surface and causes the protrusions due to the volume expansion (about 100% increase for Al to AlLi). As found from the energy dispersive X-ray spectroscopy (EDX) mappings, the intensity of Al Kα is significantly reduced at the protrusions; as Li element cannot be detected by EDX, the intensity decrease of Al Kα directly indicates a higher Li composition in such protruded regions, corresponding to the AlLi formation. It deserves to note that grain sizes of AlLi are quite different between the two cases. As found from the electron backscatter diffraction (EBSD) patterns in Fig. 2a, f, the crystalline size in Al4N-AR is far smaller than that in Al4N-HT; thus, it seems that the grain size of AlLi in each foil may reflect the crystalline size in each in the early stage of lithiation. Comparing the EDX mappings for Al4N-AR and Al4N-HT, one can find that there are light-colored areas (gray) around the protrusions (black) in Al4N-HT, but there is no such area in Al4N-AR. This indicates that, for the soft Al4N-HT matrix, lithiated regions can also grow locally starting from the protruded AlLi grains, which is strongly supported by the occurrence of large amount of dislocation slip lines observed in the SEM images.

In the further lithiation (from II to III in Fig. 2a, f), the two samples show a crucial difference in morphology. In Al4N-AR, fine AlLi particles are uniformly formed all over the surface, i.e., the homogeneous lithiation is successfully attained. On the other hand, in Al4N-HT, the AlLi phase grows preferentially from the already swelled regions by lithiation, where the Al matrix is plastically deformed with a large amount of slips. Figure 2b, c show the cross-section SEM images of Al4N-AR before and after lithiation, respectively. It is noteworthy that the very flat interface of AlLi proceeds along the depth direction from the front (i.e., facing the Li electrode) to the back side and, simultaneously, the front surface goes up to increase the foil thickness. The thickness of the lithiated layer (~80 μm) is about twice that of the initial foil (~40 μm), i.e., almost of volume expansion caused by the Al-to-AlLi transformation is realized only along the out-of-plane direction. Furthermore, by comparing Fig. 2a(II) and 2a(III), one can find that the current density would affect the nucleation frequency on the Al surface, in that a large number of AlLi protrusions are formed in (III) at 0.5 mA cm^−2^ compared with (II) at 0.05 mA cm^−2^. The unidirectional volume expansion, however, would not be affected by the current density unless the operating potential reaches the redox potential of Li^+^/Li. Figure 2d shows that the X-ray diffraction (XRD) pattern of the 53% lithiated Al4N-AR foil is in good agreement with the stardard data of AlLi crystal in the inorganic crystal structure database (ICSD). In extracting Li from the lithiated Al4N-AR foil (from III to IV in Fig. 2a), the Al matrix is self-organized to form a columnar structure with cleavages and micropores following the dealloying porous mechanism^24^, whereas in Al4N-HT foil (from III to IV in Fig. 2f), pulverization proceeds with delithiation, starting from the AlLi grains that have precedingly reached the back side of the foil, as shown in Fig. 2g, h.

### Mechanism from metallurgical insight

**Fig. 3:**
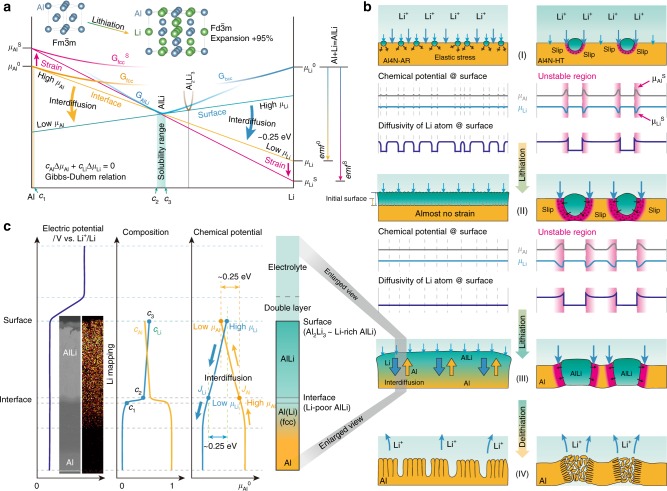
Lithiation mechanism on an Al anode. **a** The Gibbs free-energy curves in Al-Li binary system. The two-phase reaction of fcc-Al/AlLi_Li-poor_ (whose Li composition is *C*_2_) occurs during lithiation. The chemical driving force of fcc-Al/AlLi reaction (~*emf*^0^) is about 0.38 eV (yellow line), but the value is enhanced when certain amount of strain energy is accumulated by mechanical deformation (pink line). Basically, the consideration of local equilibrium of fcc-Al/AlLi_Li-poor_ is sufficient, but around the surface that of AlLi_Li-rich_/Al_2_Li_3_ is also needed during lithiation. **b** Schematic illustration of the lithiation processes of mechanically hard and soft Al foils; (left) Al4N-AR and (right) Al4N-HT. Stages (I, II, III, and IV) corresponds to the SEM images in Fig. 2a; see also Supplementary Fig. 3. The appropriate hardness of Al4N-AR enables the homogeneous lithiation. As the diffusivity in the AlLi phase possessing an ordered lattice is generally slower than that in the fcc Al phase, the formation of AlLi in the remaining Al matrix is kinetically preferential, whereas the already deformed Al matrix tends to be preferentially attacked by Li thermodynamically. **c** Schematic profiles of gradients of electric potential, composition, and chemical potentials of Li and Al to illustrate the unidirectional volume-expansion mechanism during lithiation on Al4N-AR. Insets show the SEM image and SIMS mapping of Li at the ion-beam-fabricated cross-section of a partially lithiated Al4N-AR; full field images are given in Supplementary Fig. 4. The chemical-potential gradients of Li and Al due to those of respective compositions drives the interdiffusion of Al and Li, whose maximum driving force is estimated to be about 0.25 eV.

To realize the unidirectional AlLi formation that is capable of circumventing huge volume strain, the Al element of matrix component must flow from inside to the surface during lithiation. Otherwise, the volume strain is inevitably caused by the Li compound formation, which results in significant deterioration of electrodes. Here, based on the viewpoints of thermodynamics, elasticity, and atomic diffusion, we consider the reason why such a unidirectional AlLi formation can be driven. Figure 3a illustrates schematically the Gibbs free-energy profiles in Al-Li binary system, in which the free-energy curves, *G*_fcc_, *G*_bcc_, and *G*_AlLi_, are semi-quantitatively drawn based on the thermodynamic database (fcc: Al solid solution, bcc: Li solid solution)^38^. According to the Al-Li phase diagram, the insertion of Li into Al matrix undergoes a two-phase reaction of fcc-Al/AlLi phases, where each chemical potential in the fcc Al solid solution and AlLi phase must be equal at equilibrium state; *μ*_Li in fccAl_ = *μ*_Li in AlLi_ and *μ*_Al in fccAl_ = *μ*_Al in AlLi_ as indicated by yellow line in Fig. 3a. The chemical-potential difference of Li between AlLi phase and pure Li phase determines the electromotive force (*emf*^0^ in Fig. 3a) of the lithiation reaction, Al + Li = AlLi. What we want to discuss here are the following two points: (i) in-plane homogeneous reaction of Li on the surface and (ii) transfer of Al toward the surface.

Let us first address the term (i). Difference in matrix hardness yields different strain effects during the Li compound formation. When plastic deformation of the Al matrix occurs, large amount of dislocations are introduced into the matrix, so that the Gibbs free energy of the deformed Al matrix is increased, as indicated by the *G*_fcc_^S^ in Fig. 3a. Then, according to the Gibbs–Duhem relation, *c*_Al_*dμ*_Al_ + *c*_Li_*dμ*_Li_ = 0, a two-phase equilibrium state must be shifted, as shown by a pink line in Fig. 3a. Consequently, the chemical potential of Li is apparently lowered and the electromotive force is enhanced more or less. This effect would be a main reason for the inhomogeneous lithiation in the softer Al4N-HT electrode. As soft Al matrix in the Al4N-HT electrode cannot bear the internal stress caused from the electrochemically growing AlLi phase, the volume expansion induces plastic deformation of the matrix. Afterwards, Li would prefer to be inserted into such unstable regions to obtain a chemical-potential gain. In addition, the plastic deformation involves a large amount of lattice defects, which would facilitate the Li diffusion in Al matrix. As illustrated in Fig. 3b (right), these thermodynamic and kinetic effects can work synergistically and the formation of AlLi easily reaches the back side, which leads to fracture and pulverization of electrode upon delithiation. On the contrary, as shown in the Al4N-AR foil in Fig. 1, appropriate hardness of the matrix can circumvent plastic deformation of the matrix, i.e., Li compound cannot deform the matrix. In this case, the growth of AlLi would be retarded due to the elastic stress in the Al matrix^17^. Furthermore, unlike the case of softer matrix, no preferential region for Li insertion exists in the appropriately hard matrix, so that the lithiation can evenly occur in the remaining Al matrix on the surface, resulting in the homogenous reaction in plane.

Next, we consider the term (ii) why unidirectional diffusion from the inside to surface of the Al matrix component is driven after homogeneous lithiation on the surface is completed. The inset of Fig. 3c shows SEM image and corresponding Li element mapping for the cross-section of a lithiated Al4N-AR foil, which was prepared by focused ion beam (FIB) and measured by time-of-flight secondary ion mass spectrometry (TOF-SIMS). Interestingly, it is found that Li composition in the AlLi phase slightly decreases from the surface to the inside. As the AlLi phase tolerates off-stoichiometric composition according to the Al-Li phase diagram, the composition gradient can be allowed in the AlLi single phase. Again, revisiting the Gibbs–Duhem relation, not only the chemical potential of Li but also that of Al is changed gradually, as shown in Fig. 3c; *μ*_Al_ increases, whereas *μ*_Li_ decreases from the surface toward inside. The chemical-potential difference between the surface and inside is estimated to be about 0.25 eV for each element based on the thermodynamic database^38^. This chemical-potential gradient of each element drives the interdiffusion, **J**_Al_ = −*M*_Al_ grad *μ*_Al_ and **J**_Li_ = −*M*_Li_ grad *μ*_Li_, where *M*_Al_ and *M*_Li_ are the mobilities of respective elements; thereby, Al element flows from the inside to surface, whereas Li element flows from the surface to inside. Consequently, the Al/AlLi interface gradually moves toward the inside of the Al matrix, whereas the AlLi front surface grows toward the counter-electrode side with moving the surface. This one-dimensional interdiffusion mechanism can circumvent the huge volume strain due to the lithiation. In contrast, we previously attempted to confine the volume expansion of Sn-Li alloy (most of the Sn-Li intermetallic compounds are line compounds) by embedding Sn in unidirectional porous Cu^39^, but lithiation occurred only on the Sn surface without further infiltration of Li into the Sn matrix. Thus, it is reasonably inferred that a certain tolerance to off-stoichiometry of Li intermetallic compounds is crucially important to yield the driving force of interdiffusion.

### Practical application to alloy-anode lithium battery

**Fig. 4:**
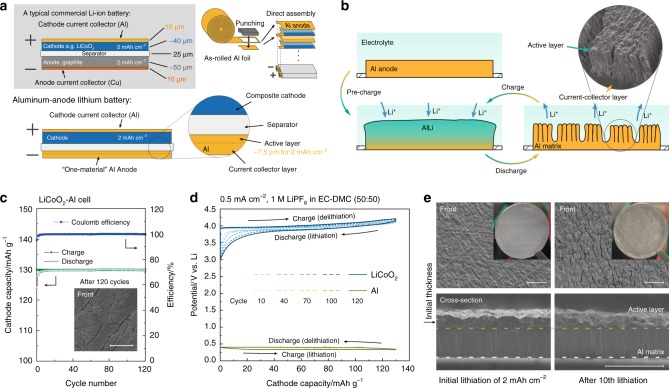
Application and performance of an Al-anode lithium battery. **a** Structure of an Al-anode Li-ion battery compared with a conventional Li-ion battery. The Cu anode current collector is not needed when using the Al anode. The as-rolled Al anode can be assembled directly. **b** Electrode reaction of an Al anode. Al matrix is partly lithiated during pre-charge and the lithiated layer forms columnar-porous structure in discharge and works as the active materials in further cycles. **c** Cycle performance of a lithium battery consisting of an Al anode and a LiCoO_2_ cathode. The inset SEM image shows the surface of the Al anode after 120 cycles. Electrode properties at initial cycles is shown in Supplementary Fig. 2e. **d** Potential profiles of cycle 10, 40, 70,100 and 120 in the cycle tests. No obvious degradation is observed on the Al anode. **e** Surfaces and cross-sections of Al anodes after initial lithiation of 2 mAh cm^−2^ and after subsequent 10th lithiation. The active layer and current-collector layer are well-retained without consuming. Scale bars in SEM images are 100 μm.

Finally, we exemplify a concept of “one-material electrode” combining active material and collector using a practical collector-free Al anode for lithium batteries. Figure 4a compares the structure of an Al-anode lithium battery to that of a conventional LIB. An Al4N-AR foil is assembled as an anode instead of the conventional graphite composite; the remaining Al matrix layer, which is indispensable to maintain the stable structure of the electrode, can also play a role of current collector and a heavy Cu foil is not needed to be employed any longer in this type of electrode, which would markedly simplify the battery manufacturing process. As shown in Fig. 4b, an Al-foil anode is partially lithiated in the pre-charge (initial lithiation) process and when Li is inversely extracted from the lithiated layer in the first discharge (delithiation) process, the remaining Al matrix shrinks to form columnar structure with producing some internal pores. This columnar-porous structure of the active material, which can be formed in-operando in the initial lithiation/delithiation cycle, plays a significant role for later cyclic charge/discharge processes. Figure 4c, d show the battery performance of a LiCoO_2_-Al cell; almost 100% Coulomb efficiency is retained and no obvious morphology change is observed after the 120th cycle. The potential-capacity profiles of the 10, 40, 70, 100, and 120th cycles are plotted in Fig. 4d, where the Al anode behaves a stable operating potential at 0.3–0.4 V vs. Li, although a slight degradation is observed on the LiCoO_2_ cathode. To confirm whether the active Al layer is maintained or consumed to fragment out, we compared the cross-section morphologies of two samples obtained after the initial lithiation of 2 mAh cm^−2^ and subsequent tenth lithiation. As shown in Fig. 4e, except a slight increase of internal spaces between the columns, the active layer substantially keeps as a bulk without fragmentation and no obvious difference is observed both in the thicknesses of active-material layer (higher part of the Al electrode) and current-collector layer (lower part of the Al electrode).

## Discussion

In the present work, the hardness of metallic foil is considered as a key parameter to balance the strengths between matrix and lithiated phase. If the strengths of both phases are comparable to each other, the high structure stability can be maintained during the first lithiation as in the case of Al4N-AR. This is because the resultant lithiated phase (AlLi) cannot deform the Al matrix and, therefore, the chemical potential of Al remains unchanged everywhere in the Al matrix. However, if there are locally deformed regions in the Al matrix, where the Al chemical potential is high (see the pink line in Fig. 3a), Li atoms preferentially attack and annihilate such unstable regions (as shown experimentally in Fig. 2f, g). Thus, the local strain induces the inhomogeneous reaction of Li with the anode material. Consequently, it is very important that the Al matrix is not deformed by the AlLi formation. Next, it is indispensable to drive the Al diffusion from matrix toward surface (interface between electrolyte/anode) to circumvent the internal volume expansion. Fortunately, it is possible for Al, because the AlLi compound tolerates the off-stoichiometric composition, so that the composition gradient can be formed in the AlLi compound. The Li composition around the surface is higher than that in the inner matrix, i.e., the Al composition is lower around the surface. Thus, the Al diffusion toward the surface can be successfully driven. Consequently, the volume expansion with lithiation is confined only to the normal direction. In terms of the tolerance of off-stoichiometric composition, Al metal is more suitable for the alloy-anode material than other elements that show stoichiometric composition in the Li compounds. However, it would be effective to add a third element for obtaining off-stoichiometric characteristics due to a certain entropy effect.

Owing to the homogenous lithiation and unidirectional volume-strain circumvention, a very flat interface is formed between lithiated phase and Al matrix (see Figs. 2c and 4e), which conceptually divides a partly lithiated Al foil to an active-material layer and a current-collector layer. Thanks to this layered structure, the one-material Al anode is endowed with an excellent structural stability. Furthermore, as the active layer once lithiated would be self-organized to form a porous structure during delithiation, the expanded volume with extra space in the columnar-porous active-material layer can accommodate the volume expansion by the subsequent lithiation. This prevents the relatively fragile AlLi from impinging with each other in further reactions and, therefore, circumvents the fatal pulverization in a similar mechanism as well as in a nanoporous silicon^24^, leading to excellent cyclability of the one-material Al anode. Consequently, focusing on the active-material layer, it shows a large capacity close to the theoretical value of ~990 ${\mathrm{mAh}}\;{\mathrm{g}}_{{\mathrm{Al}}}^{ - 1}$ and ~2680 ${\mathrm{mAh}}\;{\mathrm{cm}}_{{\mathrm{Al}}}^{ - 3}$ with quite a high cyclability. As shown in Supplementary Note 2 and Supplementary Fig. 5, benefiting from the absence of a relatively heavy Cu current collector, this type of Al anode for lithium batteries can achieve a higher practical capacity than a conventional carbonaceous anode and even than a Li metal anode (with a Cu collector), which would lead to future safe and high-energy-density lithium batteries.

## Methods

### Materials preparation

Al foils used in the present study were produced by Sumitomo Chemical Co., Ltd. As-rolled and annealed Al foils of purity 99.99% and as-rolled Al foil of purity 99.8% were prepared from corresponding Al ingots. As-rolled Zn foil of 99.9% was purchased from The Nilaco Corporation and as-rolled Sn foil of 99.9% was purchased from Takeuchi Metal Foil and Powder Co., Ltd. The electrolyte of 1 M LiPF_6_ in EC:DMC (50:50) solution was purchased from Sigma-Aldrich Co. LLC. LiCoO_2_ powder was purchased from Toshima Manufacturing Co., Ltd. Composite cathode was prepared by mixing the LiCoO_2_ powder with conductive carbon black (Super C65) and polyvinylidene difluoride binder (5% dispersed in *N*-methylpyrrolidone, Kureha Corporation) in a weight ratio of 8:1:1. The mixture was pasted on a 20 μm-thick Al foil with an adjustable film applicator (BEVS Industrial Co., Ltd) and reached a mass loading of more than 15 mg cm^−2^ of LiCoO_2_. The pasted mixture was then dried in a vacuum dryer at 120 °C for 12 h to obtain a composite cathode sheet. Li metal foil used as the counter and reference electrodes was purchased from Honjo Metal Co., Ltd.

### Electrochemical tests

Electrochemical tests were conducted by a potentiostat VMP-3 or VSP-300 (Bio-Logic SAS). Three-electrode coin-type cells (SB7, EC-frontier Co., Ltd) were assembled in glovebox filled with high-purity argon atmosphere. Working electrodes and counter electrodes had a round shape with a diameter of 16 mm. A Li metal rod with a diameter of 1 mm was used as a reference electrode. A polyethylene film or glass fiber filter was employed as the separator. The cell structure is shown in Supplementary Fig. 6.

### Morphology observation

Samples were washed by dropping tetrahydrofuran (99.5%, FUJIFILM Wako Pure Chemical Corporation) and subsequently dried in argon atmosphere. Photos of samples were taken with an iPhone Xs Max (Apple, Inc.). Microstructures of the lithiated and delithiated samples (except the inset in Fig. 3c) were observed in detail with a FE-SEM JSM-7200F (JEOL Ltd). EBSD measurement was carried out for the Al foils with a detector (Symmetry, Oxford Instruments) attached on the FE-SEM. The samples were prepared in argon atmosphere and transferred to the high vacuum chamber of FE-SEM without exposure to air. Cross-section samples in Figs. 2 and  4 were prepared with a trimming cutter (C-4, WISTA Co., Ltd). The sample in the inset of Fig. 3c was prepared using FIB to obtain a perfect cross-section plane. The subsequent TOF-SIMS measurement of Li composition was entrusted to Sumika Chemical Analysis Service, Ltd.

### Vickers hardness

Hardness of each metal foil was measured with a micro HV tester (HMV-G21DT, Shimadzu Corporation). A test force of HV0.05 (490.3 mN) was employed and hold for 15 s. More than six points were measured for each sample with appropriate intervals. The average hardness value was presented in Fig. 1.

### X-ray diffraction

XRD patterns were measured using an automated multipurpose X-ray diffractometer SmartLab (Rigaku Corporation). For the lithiated sample, an air-tight stage was used to keep an argon atmosphere throughout the experiment without exposure to air.

### Calculation of free energy

The Gibbs free energies of Al-Li binary phases shown in Fig. 3a were depicted schematically in the light of a computer calculation using the software of CaTCalc SE developed by National Institute of Advanced Industrial Science and Technology, Japan, with a thermodynamic database provided by National Institute for Materials Science, Japan.

## Supplementary information

Supplementary Information

## Data Availability

The data that support the findings of this study are available from the corresponding author upon request.
