# Supplementary material for: Circumventing huge volume strain in alloy anodes of lithium batteries
Source: Nat Commun. 2020 Apr 13;11:1584. doi: 10.1038/s41467-020-15452-0 (PMC7154030; doi:10.1038/s41467-020-15452-0)
Supplement: Supplementary file 1 — Supplementary Information [file 41467_2020_15452_MOESM1_ESM.pdf]

Supplementary Information

Circumventing Huge Volume Strain in Alloy Anodes of Lithium Batteries

Li, H. *et al*.

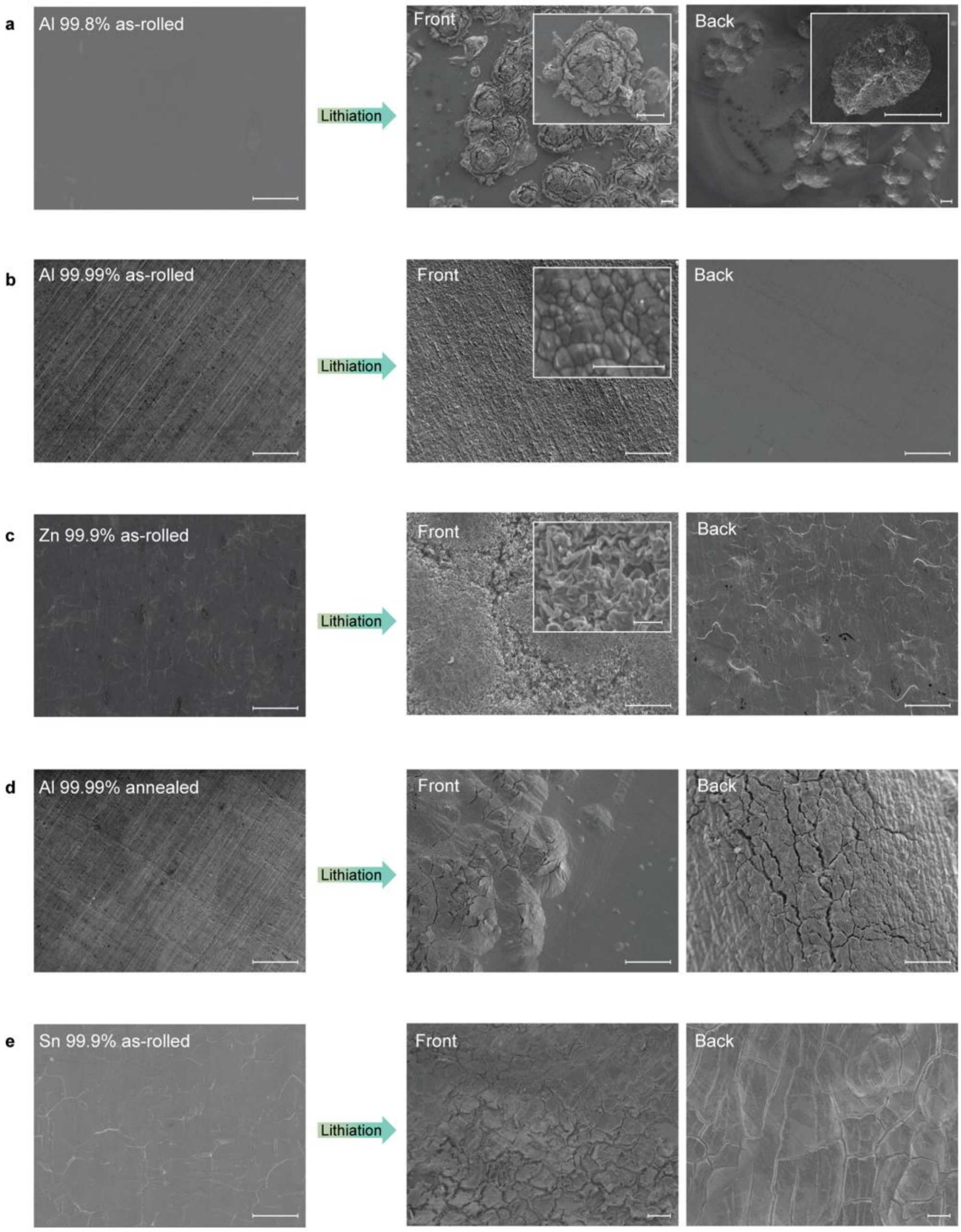

Supplementary Fig. 1 Morphology of various metal foils before and after lithiation. a, An as-rolled Al foil of purity 99.8% (Al2N-AR). Scale bar **b,** An as-rolled Al foil of purity 99.99% (Al4N-AR), **c,** An as-rolled Zn foil of purity 99.9% (Zn3N-AR) and **d,** An annealed Al foil of purity 99.99% (Al4N-HT), **e,** An as-rolled Sn foil of purity 99.9% (Sn3N-AR). Scale bars are 100 μm, except the scale bars in the enlarged view insets in **b** and **c** are 10 μm.

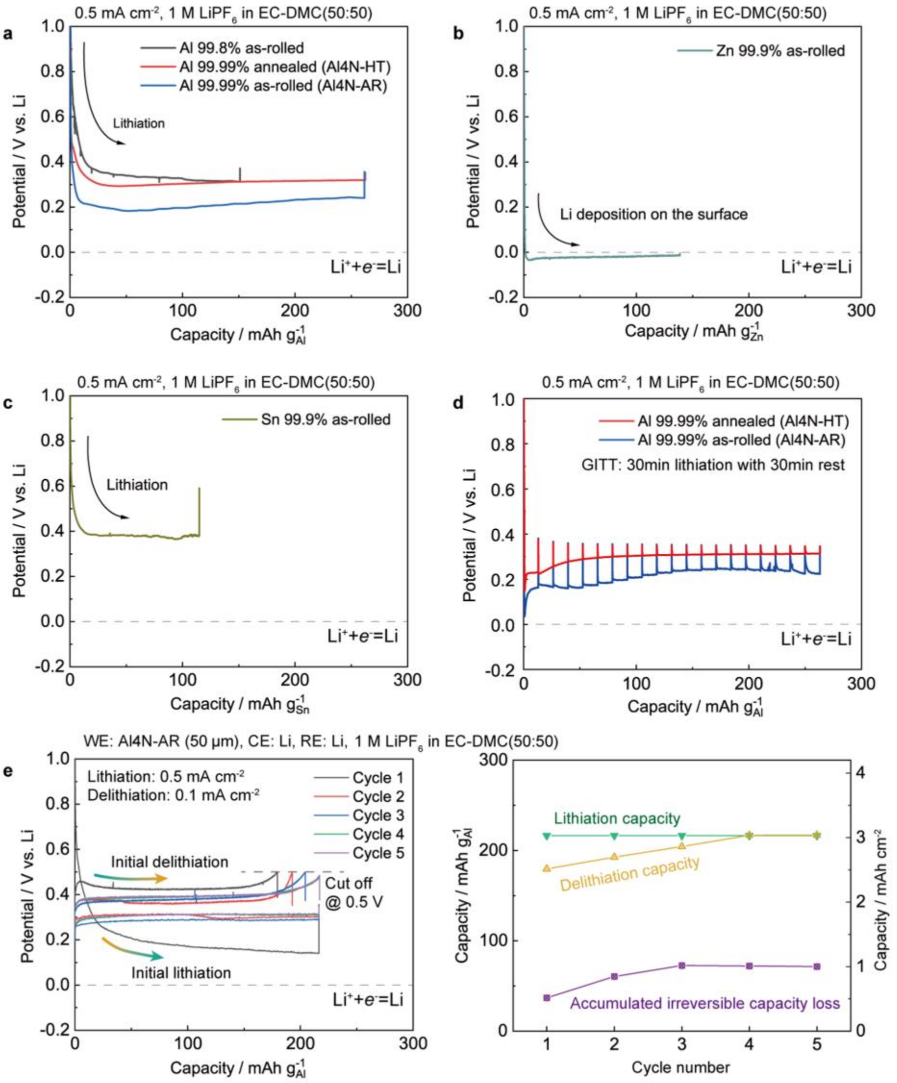

Supplementary Fig. 2 Potential profiles of various metal foils and electrode properties of Al4N-AR anode in initial cycles. Lithiation profiles corresponding to Fig.1 and Supplementary Fig. 1 are shown in **a,** Al2N-AR, Al4N-AR and Al4N-HT, **b,** Zn3N-AR and **c,** Sn3N-AR. **d,** Galvanostatic intermittent titration technique (GITT) profiles of Al4N-AR and Al4N-HT in the initial lithiation process. The Al4N-AR shows a larger overpotential than Al4N-HT due to the relatively sluggish structure relaxation in the stable structure. **e,** Potential profiles of the Al4N-AR electrode and corresponding cyclability in initial 5 cycles. Lithiation capacity of each cycle is a fixed value, and the delithiation is stopped when it is reached a cut off potential of 0.5 V vs. Li. Accumulated difference between lithiation/delithiation capacities is plotted to show the total capacity loss along with the cycles. The relatively high overpotential in cycle 1 is reduced in the further cycles due to the formation of porous structure in the active layer. The irreversible capacity in the initial cycles is suggested to be mainly caused by the Li atoms trapped in the solid-solution Al matrix and also to the SEI formation on the porous structure of the active material layer being constructed after once delithiation.

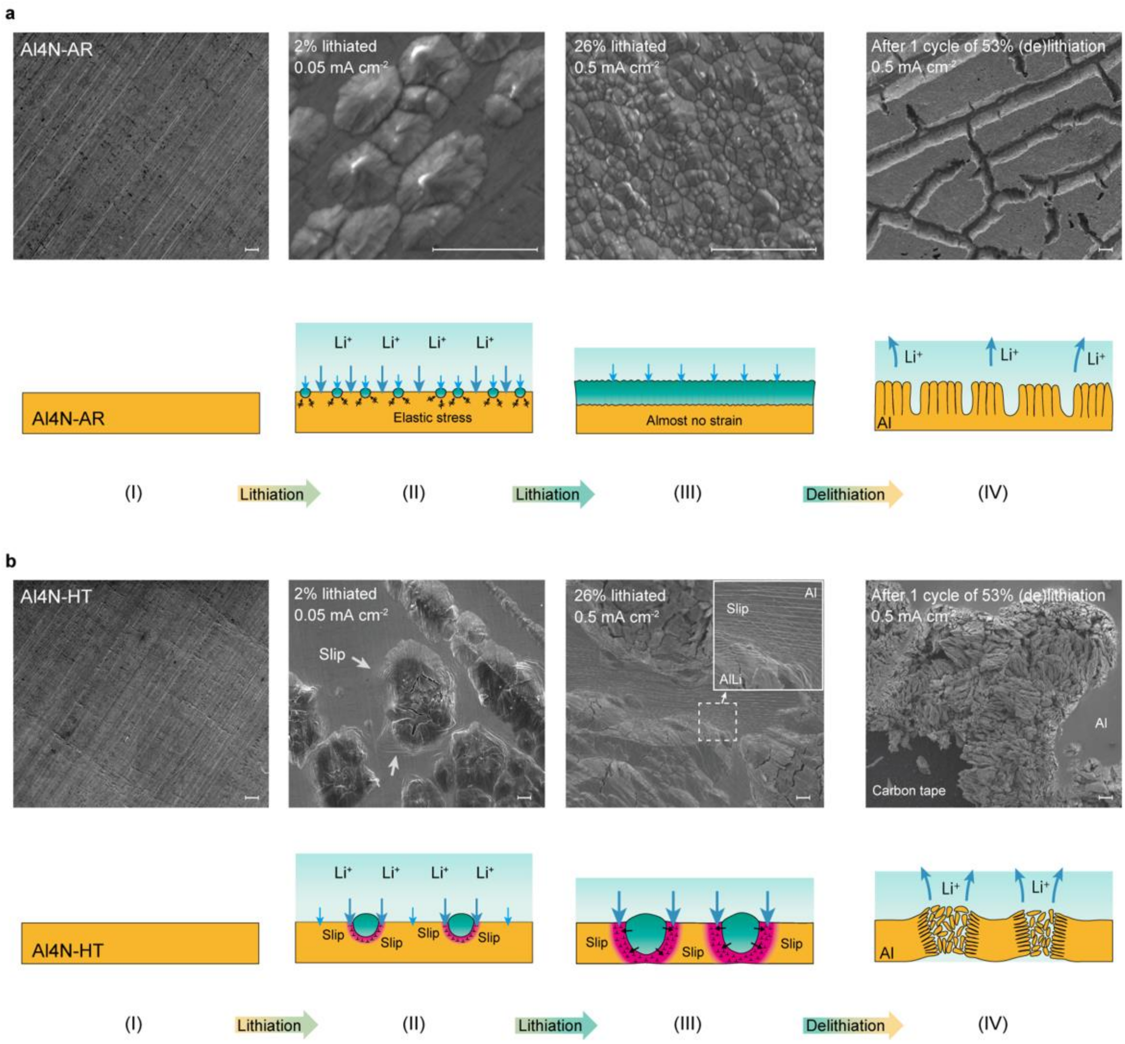

Supplementary Fig. 3 SEM images and corresponding schematic figures of initial lithiation and delithiation processes of Al4N-AR and Al4N-HT anodes. a, An Al4N-AR anode. **b,** An Al4N-HT anode. Scale bars in SEM images are 20 μm.

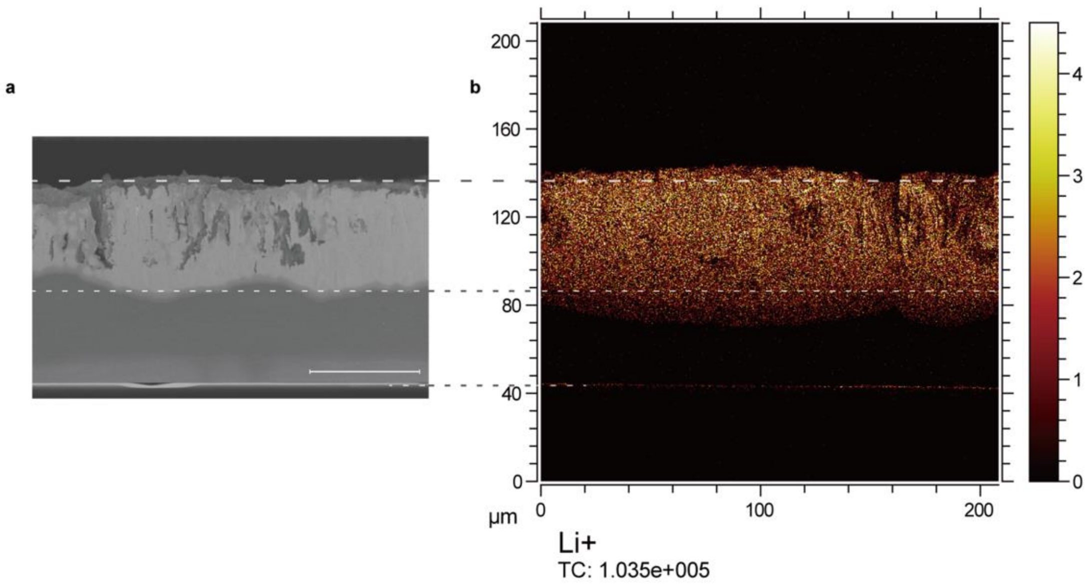

Supplementary Fig. 4 Field of SEM and TOF-SIMS observation of a lithiated Al anode. Cross sections of a lithiated Al4N-AR anode was prepared by Focused Ion Beam (FIB). The Al anode underwent 5 charge-discharge cycles in a $LiCoO_2$-Al two-electrode coin cell (CR-2032) with 1 M $LiPF_6$ in EC-DEC(30:70) electrolyte. **a,** A SEM image of the cross section. Scale bar is 50 μm. **b,** Li composition on the cross section observed by TOF-SIMS. Note that, due to the technique issue, it is not possible to observe the exact same field in both SEM and SIMS.

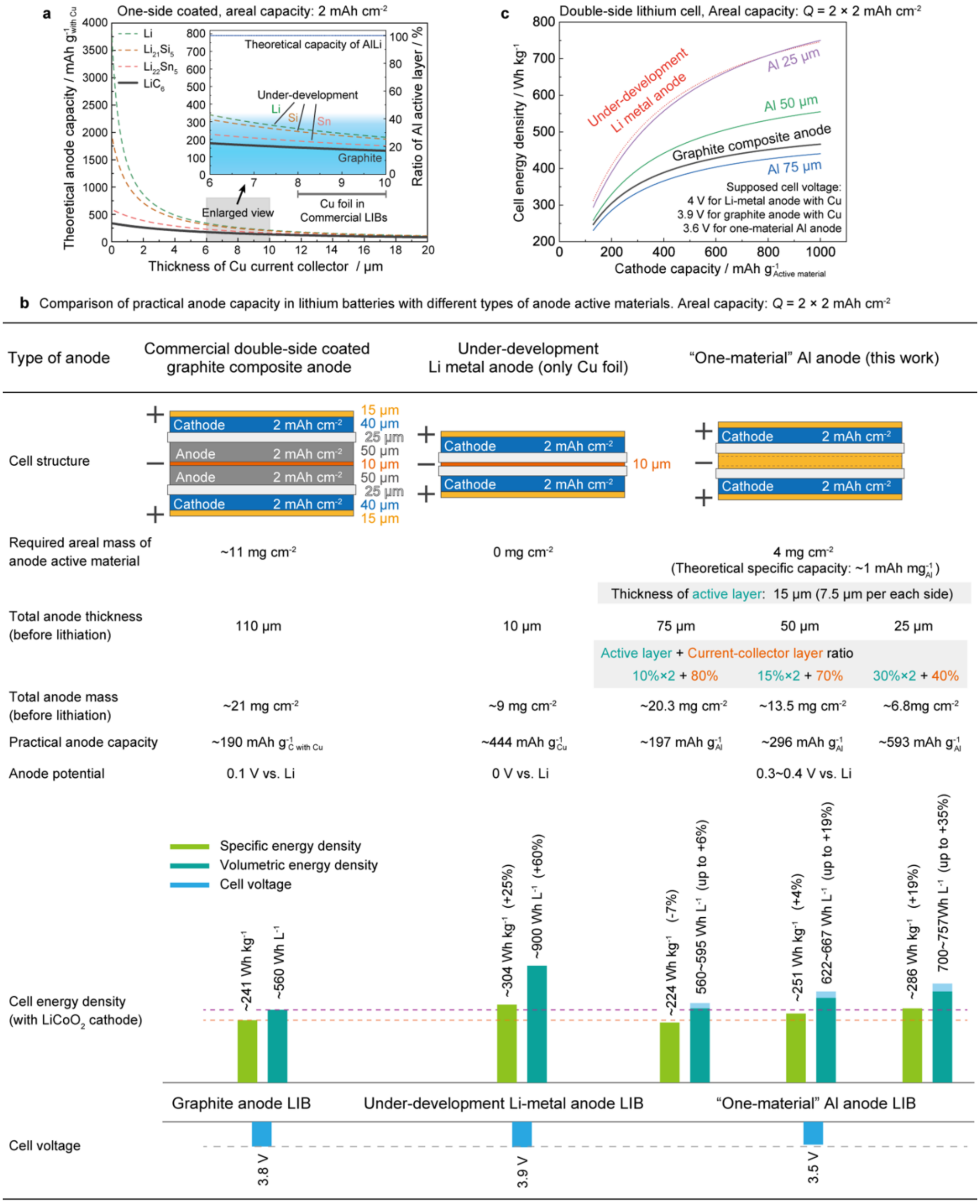

Supplementary Fig. 5 Comparison of the practical anode capacity and cell energy density. a, The total capacities of Li, $Li_{21}Si_5$, $Li_{22}Sn_5$, LiC_6 anodes depending on the thickness of Cu current collector. It is assumed that one side of the Cu foil is used. **b,** Comparison of practical capacity of a commercial graphite anode, Li metal anode and the Al anode (this work). A double-side anode is assumed to simulate the situation of a laminate-type cell. Bar graph compares the energy densities of the cells using a $LiCoO_2$ cathode, which are estimated based on the illustrated cell structures. Note that the cell voltage is given by taking account of each anode potential. The energy-density increases of respective cells are also given compared to the graphite-anode LIB. **c,** Dependence of cell energy density on cathode-active-material capacity. The cathode potential is fixed at 4 V vs. Li in evaluating of the cell-energy density.

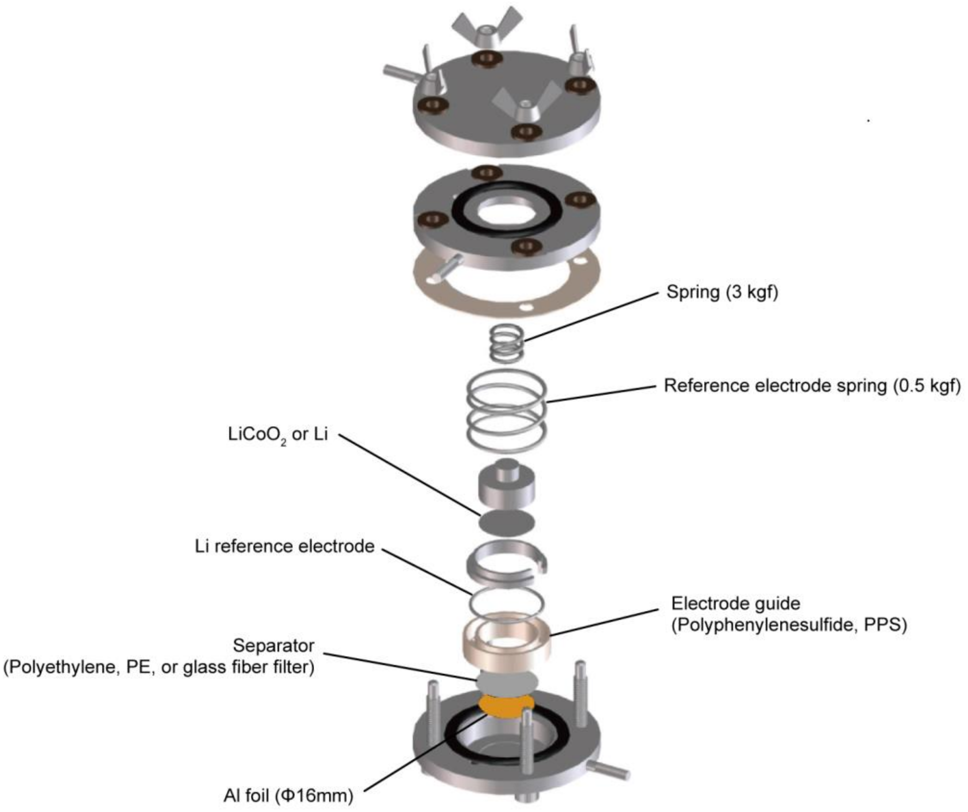

Supplementary Fig. 6 Structure of the three-electrode coin-type cell. The cell produced by EC-frontier Co. Ltd. was used in the present study. We obtained permission to use the cell construction figure.

Supplementary Note 1. Mophology and potential profiles in initial lithiation

As-rolled Al of purity 99.8% (Al2N-AR). As shown in Supplementary Fig. 1a, on the front side of Al2N-AR, lithiated phase is crushed like cabbages, indicating a brittle fracture of growing AlLi grains. On the other hand, dimples are observed on the back side, which suggests that a ductile fracture occur in the Al matrix during lithiation. Compared to the other Al4N samples, Al2N-AR shows a low overpotential in the initial lithiation process (see Supplementary Fig. 2a). This would be attributed to the fast structure degradation or relaxation only near the surface, where the formed AlLi phase is crushed by the Al matrix, leading to its pulverization.

As-rolled Zn foil of purity 99.9% (Zn3N-AR). Although Zn can form several intermetallic compounds with Li according to the binary phase diagram, the practical lithiation seems to be difficult to occur in a practical LIB cell. The electrode potential shown in Supplementary Fig. 2b reaches below 0 V vs. Li immediately on lithiating, which is consistent with the deposition of pure Li dendrites observed on the Zn electrode surface.

As-rolled Sn foil of purity 99.9% (Sn3N-AR). Since the lithiation potential in Supplementary Fig. 2c is slightly lower than ~ 0.4 V vs. Li, various compositions of Li_xSn ($x \leq 4.4$) compounds would be formed simultaneously in the Sn matrix.

As-rolled and heat-treated Al of purity 99.99% (Al4N-AR and Al4N-HT). During initial lithiation, the soft Al4N-HT shows an inhomogeneous lithiation behavior and the Al matrix deformed plastically. In contrast, the Al4N-AR shows a homogeneous lithiation behavior as well as a high structural stability. For the Al4N-AR, volume relaxation during lithiation can only be released by driving the interdiffusion of Al and Li between matrix and surface. Therefore, high overpotential occurs compared to other Al foils whose volume relaxation can be released by fracture or deformation.

Supplementary Note 2. Evaluation of capacity and energy density of a cell with "one-material" Al anode. First of all, we consider the effect of Cu collector weight on the total gravimetric capacity of anode. To do so, we set that the areal capacity of the anode consisting of active material and Cu collector is 2 mAh cm^{-2}; this areal capacity is frequently used in the commercial Li ion batteries. The active material mass required for the areal capacity is given by dividing 2 mAh cm^{-2} by the theoretical capacity of the active material. The total mass is given by a sum of the active material mass and Cu collector mass. Supplementary Fig. 5a shows the

whole gravimetric capacity of various anode materials, such as Li, powder C, powder Si, powder Sn (plate shape would not be feasible), as a function of thickness of the Cu collector.

The practical capacity of a Li-ion battery (LIB) depends on the capacity of electrode materials and other supporting materials, such as current collectors and separator infiltrated by electrolyte. Thus, it is not simple to directly compare the practical capacities of batteries with different cell structures. To facilitate the comparison, here we assume “model Li cells” consisting of a double-side anode and two composite cathodes, which are coated one-side, as shown in Supplementary Fig. 5b. The cell capacity, C, of such a double-side lithium cell can be given by the follow equation:

$$C[\text{mAh g}^{-1}] = \frac{Q[\text{mAh cm}^{-2}]}{M_\text{C}[\text{g cm}^{-2}] + M_\text{A}[\text{g cm}^{-2}]} = \frac{1}{\frac{M_\text{C}}{Q} + \frac{M_\text{A}}{Q}} \quad (1)$$

M_C and M_A are areal masses of the cathode and anode, respectively, in the illustrated cell in Supplementary Fig. 5b, which are sum of both the mass of active materials and supporting materials, such as current collector and binders. Note that masses of separator and electrolyte are ignored in this equation. Q denotes an areal capacity of the double-side Li cell. To facilitate the comparison, here we assume a fixed areal capacity of 4 mAh cm^{-2} for the cell (Q = 4 mAh cm^{-2}), which means that an areal capacity of one side of the electrodes is 2 mAh cm^{-2}. $C_\text{C}\ (= Q/M_\text{C})$ and $C_\text{A}\ (= Q/M_\text{A})$ are the actual capacity of the cathode and anode, respectively; see the cell structure in Supplementary Fig. 5b.

As an example, a $LiCoO_2$/Carbon/PVDF = 90/5/5 composite cathode is assumed for calculating capacity and energy density. When the capacity of $LiCoO_2$ is set at 130 mAh g^{-1}, the required areal mass of the $LiCoO_2$ active material is 2 mAh cm^{-2} / 130 mAh g^{-1} = 15.4 mg cm^{-2}. When the thickness of cathode current collector (Al foil) is assumed to be 15 μm, the mass is ~ 4 mg cm^{-2} (Al density: 2.7 g cm^{-3}). Thus, the total mass of such a one-side composite cathode is given by 15.4 mg cm^{-2} / 90% + 4 mg cm^{-2} = 21.1 mg cm^{-2}.

As for the conventional double-side coated graphite anode, a graphite/PVDF (90:10) composite anode with a 10-μm-Cu current collector is assumed; the 10-μm-Cu foil is most widely used in commercial LIBs. Mass of graphite can be calculated by diving the areal capacity Q by the theoretical capacity, 372 mAh g^{-1}, and the result is about 10.8 mg cm^{-2} for a double-side coated anode. For the Cu foil, the areal mass is about 9 mg cm^{-2}, which is obtained by multiplying the thickness (10 μm) and the density of about 9 g cm^{-3}. Therefore, the total mass of the double-side

graphite anode is about 10.8 mg cm^{-2} / 90% + 9 mg cm^{-2} = 21 mg cm^{-2}, and the actual anode capacity, C_A, is 4 mAh cm^{-2} / 21 mg cm^{-2} ≈ 190 mAh g^{-1}.

For a Li metal-anode cell, the highest capacity can be obtained when there is no excess Li on the anode (in this case, all Li is from the composite cathode). The mass of the Cu current collector (9 mg cm^{-2}) would be the total mass of the double-side anode. Therefore, the actual anode capacity, C_A, is 4 mAh cm^{-2} / 9 mg cm^{-2} ≈ 444 mAh g^{-1}.

In the one-material Al anode concept in this work, a higher practical capacity can be obtained by using an Al foil with a thinner current collector layer. For a certain areal capacity, the thickness of active layer is obtained by dividing the areal capacity Q by the theoretical volumetric capacity of Al (2680 mAh cm^{-3} for Al+Li = AlLi). Thus, a 15-μm-thick (≈ 4 mAh cm^{-2} / 2680 mAh cm^{-3}) active layer is required for the double-side Al anode (i.e., 7.5 μm per each side). The actual capacities of 75 μm, 50 μm and 25 μm Al foils are provided as examples in Supplementary Fig. 5b. For instance, the mass of a 75-μm-thick Al foil is 75 μm × 2.7 g cm^{-3} ≈ 20.3 mg cm^{-2}, as given in Supplementary Fig. 5b. Thus, C_A of the 75 μm Al anode is 4 mAh cm^{-2} / 20.3 mg cm^{-2} ≈ 197 mAh g^{-1}, which is very close to that of a commercial double-side coated graphite anode. The ratio of active material layer in the 75-μm Al anode is 15/75 = 20% (i.e., 10% per each side), that is, about 80% of the Al foil remains as a current collector layer.

The cell energy densities shown in Supplementary Fig. 5b are calculated by assuming operating potential of $LiCoO_2$ to be 3.9 V vs. Li. Thus, the cell voltage for a graphite anode cell would be 3.8 V, and that for an Al anode cell would be about 3.5 V. Especially for the one-material Al anodes, the volumetric energy densities are calculated for the two typical cell volumes: the maximum (fully expanded state by the lithiation) and minimum (initial state before lithiation) volumes. Moreover, in order to show the dependence of cell energy density on cathode-active-material capacity, we suppose a cathode active material showing 4 V vs. Li as an operating potential, the results of which are shown in Supplementary Fig. 5c. One can find that a marked increase of the cell energy density is obtained for the “one-material” Al-anode lithium batteries, if a high capacity cathode material would be available.
